# In Silico Modeling of Fabry Disease Pathophysiology for the Identification of Early Cellular Damage Biomarker Candidates

**DOI:** 10.3390/ijms251910329

**Published:** 2024-09-25

**Authors:** Javier Gervas-Arruga, Miguel Ángel Barba-Romero, Jorge Julián Fernández-Martín, Jorge Francisco Gómez-Cerezo, Cristina Segú-Vergés, Giacomo Ronzoni, Jorge J. Cebolla

**Affiliations:** 1 Takeda Development Center Americas Inc., Cambridge, MA 02142, USA; javier.gervas@takeda.com; 2 Department of Internal Medicine, Albacete University Hospital, 02006 Albacete, Spain; mabarbar@sescam.jccm.es; 3 Albacete Medical School, Castilla-La Mancha University, 02006 Albacete, Spain; 4 Department of Internal Medicine, University Hospital Álvaro Cunqueiro, 36312 Vigo, Spain; julianfernandezmartin58@gmail.com; 5 Department of Internal Medicine, Infanta Sofía University Hospital, 28702 Madrid, Spain; jfrancisco.gomez@salud.madrid.org; 6 Faculty of Medicine, European University of Madrid, 28670 Madrid, Spain; 7 Anaxomics Biotech S.L., 08007 Barcelona, Spain; cristina.segu@anaxomics.com; 8 Takeda Farmacéutica España S.A., 28046 Madrid, Spain; giacomo.ronzoni@takeda.com

**Keywords:** alpha-galactosidase A, Fabry disease, machine learning, systems biology, biomarkers

## Abstract

Fabry disease (FD) is an X-linked lysosomal disease whose ultimate consequences are the accumulation of sphingolipids and subsequent inflammatory events, mainly at the endothelial level. The outcomes include different nervous system manifestations as well as multiple organ damage. Despite the availability of known biomarkers, early detection of FD remains a medical need. This study aimed to develop an in silico model based on machine learning to identify candidate vascular and nervous system proteins for early FD damage detection at the cellular level. A combined systems biology and machine learning approach was carried out considering molecular characteristics of FD to create a computational model of vascular and nervous system disease. A data science strategy was applied to identify risk classifiers by using 10 K-fold cross-validation. Further biological and clinical criteria were used to prioritize the most promising candidates, resulting in the identification of 36 biomarker candidates with classifier abilities, which are easily measurable in body fluids. Among them, we propose four candidates, CAMK2A, ILK, LMNA, and KHSRP, which have high classification capabilities according to our models (cross-validated accuracy ≥ 90%) and are related to the vascular and nervous systems. These biomarkers show promise as high-risk cellular and tissue damage indicators that are potentially applicable in clinical settings, although in vivo validation is still needed.

## 1. Introduction

Fabry disease (FD) (MIM#301500) is considered as inborn error of glycosphingolipid metabolism due to reduce to null catalytic activity of the acid hydrolase α-galactosidase A (α-Gal A) (EC 3.2.1.22), which results in the accumulation of globotriaosylceramide (Gb3), globotriaosylsphingosine (lyso-Gb3), and other glycosphingolipids (such as galabiosylceramide, Gb2, etc.) in different tissues [1]. The disease is caused by pathogenic variants in the *GLA* gene (MIM*300644). Currently, nearly 1000 genetic variants have been described in relation to FD [e.g., NM_000169.3(GLA):c.713G>A (p.Ser238Asn), NM_000169.3(GLA):c.847C>T (p.Gln283Ter), etc.] [2]. The inheritance pattern of the disease is X-linked; therefore, all males carrying one pathogenic allele manifest symptoms of the disease [1]. In contrast, females carrying one or two alleles may exhibit a variable clinical presentation due to different X-chromosome inactivation profiles in different organs [3]. The estimated incidence and prevalence of FD have increased in recent years to 1:117,000 and 1:8882 [4,5], respectively, or even more, mainly through the detection of variants of unknown significance or associated with nonclassical or classical phenotypes in newborn screening [6,7,8].

FD has two phenotypic forms of presentation: a classical phenotype and a nonclassical phenotype [9]. The classical form of the disease usually begins in childhood, with earlier involvement in males, while the nonclassical phenotypic condition is characterized by a more variable disease course in which patients are generally less affected initially and manifestations present later, usually during adulthood, and are often confined to a single affected organ [9,10]. The clinical recognition of these mono-organic forms is crucial in the journey of FD patients, as they do not present the plethora of characteristic signs and symptoms of the disease and because there are phenocopies with higher prevalence at the cardiac level (e.g., hypertrophic cardiomyopathy [11]) and renal level. The latter pose a challenge since the phenotypic spectrum can overlap with entities of both non-genetic origin (e.g., IgA or IgM nephropathy) and genetic origin (e.g., Alport syndrome, polycystic kidney disease, or autosomal dominant polycystic kidney disease, among others [12]). For this reason, genetic screening of FD in nephropathies of unknown origin is particularly relevant today [13].

The most characteristic early clinical symptoms in patients with classical FD, especially in the first years of life, are hypo- or anhidrosis and recurrent burning and shooting pain in the distal extremities, which is associated with peripheral neuropathy. Patients usually suffer from a decreased sensation of heat and cold and frequent gastrointestinal disturbances [14,15,16]. Vascular involvement can lead to the development of disease at the cardiac, kidney, and central nervous system (CNS) levels and to cerebrovascular events such as a stroke or transient ischemic attack [17,18,19]. On the other hand, there is a high proportion of patients with a greater risk of developing neuropsychiatric symptoms, such as depression and neuropsychological deficits [20]. Late complications and disease progression can eventually cause significant morbidity and mortality [21].

The pathogenesis of FD is related to α-Gal A deficiency. The absence of this enzymatic activity causes systemic accumulation endogenously synthesized Gb3, as well as that sourced from the autophagocytosis of cellular membranes, and its deacylated derivative lyso-Gb3, leads to significant cellular alterations [22]. At the cellular level, this buildup of undegraded lipid material disrupts the physiological processes of endocytosis and autophagy, induces apoptotic mechanisms, causes mitochondrial dysfunction, and disturbs endoplasmic reticulum homeostasis [23]. These disruptions result in inflammatory processes, oxidative stress, and fibrosis that trigger the entire pathophysiology of this disease [20,24]. Fundamentally, the primary cellular types involved in this accumulation are endothelial cells, epithelial cells, pericytes, myocardial, ganglion, and smooth muscle cells [1]. Therefore, the main pathophysiological alterations in FD are vascular dysfunction involving the accumulation of glycosphingolipids, structural alterations of vessel walls, the activation of the endothelium by the release of proinflammatory molecules, and the occurrence of a prothrombotic state [25]. The physiopathological basis of the nervous system needs to be better understood. In the CNS, there is cerebral angiopathy. In the peripheral nervous system, the accumulation of glycosphingolipids in Schwann cells and dorsal spinal ganglia seems to affect A-delta and C fibers, which is represented by neuropathic pain and by autonomic alterations. The classical neurological manifestations of FD are neuropathy and premature cerebrovascular disease [26]. However, in addition to vascular or neuropathic complications, FD can lead to the development of psychiatric and cognitive disorders [27,28]. Depression is common, while acute psychotic symptoms, personality and behavior changes, or sleep disturbances may appear less frequently [27,28,29].

Early diagnosis is essential to avoid irreversible organ damage and to promptly apply therapeutic options that can reduce patient morbidity and mortality. In contrast, late disease detection is associated with a lower response to treatment [30,31]. Real-world data (mainly disease registries) reveal that early enzyme replacement therapy (ERT) could delay progressive organ damage and, therefore, improve patients’ quality of life. Currently, early diagnosis through newborn screening is not universally accepted, primarily due to ethical considerations (e.g., the large number of neonates identified with late-onset or uncertain significance genetic variants) [32]. Early diagnosis should be considered in well-recognized risk groups or in relatives of diagnosed probands to prevent the onset of signs and symptoms of the pathology and to improve the prognostic profile by facilitating early therapeutic intervention [33]. At present, there are no established early diagnostic tools without incurring ethical limitations, therefore the discovery of early damage biomarkers for FD is an urgent need for the early identification, follow-up, and treatment of patients, and for correct prevention of irreversible damage [30,31,34].

Although much of the research effort has focused on the identification of new biomarkers, lyso-Gb3, the deacylated form of Gb3, has been established as a diagnostic biomarker and for patient stratification in FD; however, the clinical significance of the extent of changes in lyso-Gb3 and its correlation with clinical outcomes are yet to be determined. Therefore, it is advisable to monitor additional nonspecific biomarkers of FD [35,36]. These nonspecific biomarkers of FD indicate the specific damage to an organ and help measure the progression of the disease or the therapeutic efficacy of treatment. For instance, proteinuria, especially albuminuria, allows the assessment of early kidney damage, whereas the glomerular filtration rate is useful for the assessment of advanced kidney injury. The other helpful approaches are the use of CNS imaging biomarkers and the lack of follow-up symptoms in patients with FD [37]. Owing to a vital clinical need, it is necessary to identify biomarkers capable of detecting early vascular and neurological damage, as early clinical events, before irreversible alterations occur in organs at late stages.

Computational modeling and systems biology methods are increasingly implemented to help understand the molecular determinants of certain diseases, clarify disease staging, and aid drug discovery [38]. Specifically, in the field of rare disease research [39,40,41], where few data are available, integrative approaches can help open new lines of research and advance clinical management. The Therapeutic Performance Mapping System^®^ (TPMS) technology [42] uses pattern recognition and machine learning techniques to integrate available biological, pharmacological and medical knowledge and create mathematical models that simulate disease phenotypes in silico. The models are built around a complex network of proteins and their known relationships (either physical, biochemical, or regulatory) and allow us to explore the predicted protein activity changes that are induced by altered states (e.g., a pathological state or drug treatment) [42,43]. This method allows modeling of the pathophysiology of a disease and of possible cellular changes that induce irreversible tissue damage to identify biomarkers that are likely to precede the damage. Through these models, it could be possible to evaluate the involvement of proteins in little-studied manifestations or poorly accessible in vivo tissues, as is the case for neurological manifestations in FD. The results obtained through this technology have been previously proven useful for the exploration of drug mechanisms [44,45,46] and the identification of biomarkers [47,48].

In silico modeling therefore offers an alternative approach that may help identify protein biomarker candidates for the early identification of and follow-up on FD comorbidities. Accordingly, the aim of this study was to develop and evaluate a FD model to identify biomarkers associated with cellular changes involved in the initiation and progression of organ damage. We also aimed to identify candidate proteins related to vascular alterations, whose implication is characteristic of the disease, and unexplored neuronal involvement. Therefore, we applied the TPMS technology to model FD and used a data science strategy to generate hypotheses for the identification of biomarkers that can detect early damage and could be applied in clinical practice.

## 2. Results

### 2.1. Fabry Disease Characterization and Model Creation

**Figure 1:**
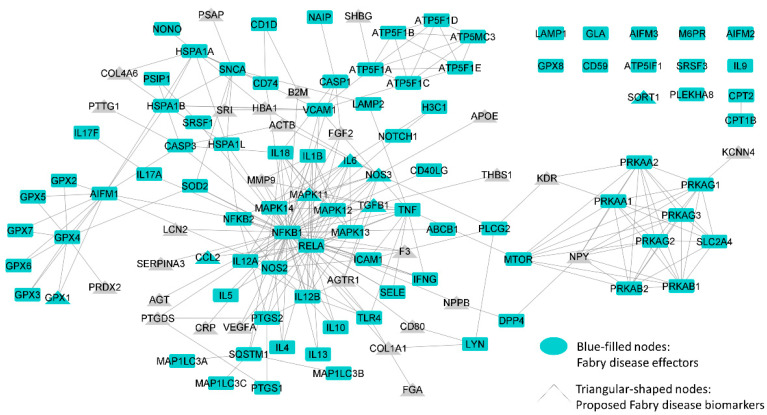
Graphical representation of Fabry disease protein network, including all effectors and direct relationships among them and previously proposed biomarkers that are directly related to them. Image was created with Cytoscape version 3.8 [49].

We analyzed 92 unique protein effectors (Supplementary Table S1) involved in FD according to the available literature data that were contextualized in human physiology through their integration into the global human protein–protein interaction network. In addition to identifying protein effectors through a literature search, we identified several proteins and nonprotein biomarkers that have already been established or previously proposed for FD (Supplementary Table S2, Figure 1).

Next, we applied the TPMS technology to create FD models. First, we explored which protein effectors, in addition to α-Gal A deficiency, were able to induce the disease according to the TPMS models and found that the nuclear factor NF-kappa-B p105 subunit, histone H3.1 and caspase-3 were able to induce at least 75% of the disease definition in terms of predicted protein activity signals. Thus, we used these four proteins as stimuli to create FD models. The model obtained contained 1011 proteins modulated on at least one of the 1000 solutions calculated.

We considered the model-derived signal of FD (based on the predicted protein activity of each of the FD effectors) in each solution as a proxy of the amount of cellular alterations and selected the top 10% and the lower 10% of the solutions to create high- and low-damage models, respectively.

### 2.2. Results of the Data Science Strategy

Considering the proteins present in the models, 1011 predicted protein activities and 1,021,110 pairwise combinations were evaluated to identify classifiers that were able to separate high- versus low-damage FD models.

For this purpose, we first evaluated the already described protein biomarkers to set a threshold (Table 1). By ranking these markers based on the cross-validated accuracy, we selected the first easily measurable candidate, β-2-microglobulin (B2M), which is a plasma protein. Thus, 70.5% was set as the cross-validated accuracy. When evaluating the proteins present in the model, we found 162 classifiers (two of which were composed of two variables) with a higher cross-validation accuracy than that (70.5%) obtained for B2M (Supplementary Table S3).

### 2.3. Biological and Clinical Filtering of the Candidates

From these 162 candidates, we selected 61 candidates that have been reported to be measurable in plasma and/or urine to focus on candidates with greater promise for translation to clinical practice (Supplementary Table S3). Finally, 36 biomarkers related to vascular and/or neuronal locations were selected (Table 2).

**Figure 2:**
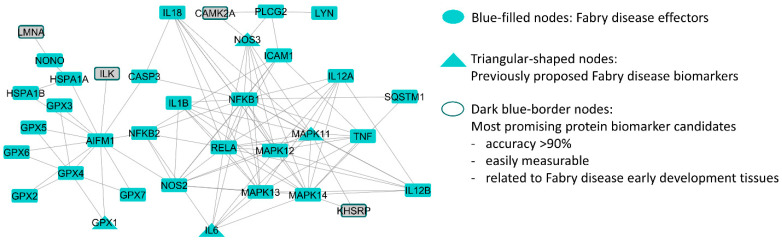
Graphical representation of the most promising biomarker candidates and their direct relationships with Fabry disease effectors according to the protein–protein interaction network. Image was created with Cytoscape version 3.8 [49].

Among them, the interleukin-18 protein was described as an effector of FD during its molecular characterization (Table 2). With a cross-validation accuracy of ≥90%, we identified calcium/calmodulin-dependent protein kinase (CAMK2A), integrin-linked kinase (ILK), lamin A (LMNA), and KH-type splicing regulatory protein (KHSRP). All of them are measurable in plasma, and only lamin A is also measurable in urine (Table 2). These proteins are directly connected to the FD protein network (Figure 2).

## 3. Discussion

The results of this in silico study show that the application of tools based on systems biology and machine learning, such as the TPMS, could help identify potential biomarkers for the early detection of damage in FD that are related to the vascular and nervous systems and allow noninvasive measurement and easy transfer to clinical practice.

The most widely used biomarker for FD to date is lyso-Gb3, which has a good utility in FD diagnosis. However, only a limited number of studies have demonstrated the correlation between lyso-Gb3 concentrations and clinical manifestations of the disease [35,36]. Therefore, more studies are needed to identify precise markers of the disease that could be used before irreversible damage occurs [54]. Moreover, the isoforms and/or analogs of Gb3 and lyso-Gb3 could be considered good candidates for the diagnosis of high-risk populations [55]. However, there are no very specific biomarkers for the responses to vasculopathy, nephropathy, and/or cardiomyopathy, which are involved in the pathogenic processes that trigger chronic inflammatory pathways and the development of irreversible fibrosis [56]. Biomarkers should be evaluated with caution since they do not allow the identification of all the phenotypes and systems affected by the disease.

From a systemic point of view, the following biomarkers have been used to assess the effectiveness of ERT and organ damage in FD: inter α-trypsin inhibitor heavy chain 4 (an anti-inflammatory protein), serum amyloid A1 (an acute-phase protein), enolase 1 (a tissue remodeling factor indicating the contribution of ischemic vascular pathology), and the enzyme endothelial nitric oxide synthase [57,58]. Meanwhile, microRNAs (miR21, miR210, miR29, miR200, miR21-5p, miR19a-3p, and miR184) [59,60] or circular RNAs [61], although they have diagnostic and prognostic utility, are used only for research purposes. Nonspecific molecular and imaging biomarkers of FD are widely used to assess kidney, heart, and blood vessel damage [37]. In the case of renal function, the following parameters are used: proteinuria, albuminuria, estimated glomerular filtration rate, uromodulin, N-acetyl-β-D-glycosaminidase and B2M [62,63]. For cardiac activity and vascular function, 3-nitrotyrosine [64,65,66], tumor necrosis factor (TNF), interleukin-6, and TNF receptors 1 and 2 are used [67,68]. Despite the availability of all the various biomarkers, few are useful for detecting early damage before irreversible tissue alterations occur in patients with no obvious manifestations of FD.

Our findings suggest some candidates that may be associated with the first signs and symptoms of the disease in vascular or neuronal tissues and could improve disease monitoring. In total, we identified 61 candidate biomarkers that can be measured in body fluids and have greater classification potential than the best previously proposed biomarkers (Table 1). One such example is B2M, being a light chain major histocompatibility class, present on the cell surface of nucleated cells. During cellular rearrangements, B2M detaches from the heavy chain and circulates in the bloodstream until it is filtered by the glomerulus. Increased levels of B2M in urine are indicative of early proximal tubular dysfunction [69] and show a significant correlation between plasma and urine concentrations, as well as the progression of renal disease in patients with FD [70]. In the classical phenotype of FD, the cardiovascular system and neuronal involvement are essential for the development of the disease and for the understanding of the clinical situation of patients. In our study, we carried out an exploratory evaluation to detect biomarkers related to vascular involvement and those that fit into the little-known field of the nervous system. Thirty-six biomarker candidates were identified to be related to vascular or neuronal locations, which are tissues affected early by FD, and thus have the potential to reflect early disease-related changes in these areas. We further highlighted candidates that have been previously functionally associated with FD development (interleukin-18, with great cardiovascular involvement [71]) and those with high classification potential (accuracy ≥ 90%) related to both vascular and neuronal tissues (CAMK2A, ILK, LMNA, and KHSRP).

These four biomarkers are connected to the effectors of FD. ILK and LMNA could be related to vascular manifestations observed in FD. ILK is an integrin-linked kinase involved in the conformational activation of the lymphocyte function-associated antigen-1 complex, which is responsible for the adhesion and extravasation of neutrophils, primarily by recognizing the intercellular adhesion molecule 1 (ICAM-1) present on the vascular endothelium. Deficient expression of ILK leads to suppresses chemokine-induced extravasation of neutrophils and ischemia-induced reperfusion injury [72]. LMNA is a protein that forms part of the nucleoskeletal intermediate filaments, playing a multifaceted role in cellular functions. These functions include maintaining the structural integrity of the nucleus, regulating gene expression, and facilitating mechanosensing and mechanotransduction via lamina-associated proteins. [73]. From the pathophysiological point of view, vascular pathogenesis in FD is related to the accumulation of glycosphingolipids [24]. This process produces a cascading effect, causing endothelial dysfunction and altered arterial remodeling, as well as occlusive phenomena and thrombosis in the medium and long terms [74]. ILK promotes monocyte adhesion and extravasation, which may lead to endothelial dysfunction and trigger inflammation and subsequent fibrosis, as observed in the previously mentioned vascular phenomena in FD [75]. In addition, the deposition of Gb3 in myocardial cells and the alteration of the expression of adhesion molecules promote inflammation, increased deposition in the extracellular matrix, and fibrosis [76]. Dysfunctional LMNA causes defects in the nuclear lamina, which disrupts mechanotransduction and mechanosensing in cardiomyocytes. This disruption leads to changes in gene expression and cellular dysregulation, affecting structures from the nuclear lamina to cell-cell junctions, and significantly contributing to the development of cardiomyopathy as observed in FD [73]. Therefore, ILK and LMNA could be involved in the pathophysiology of FD. On the other hand, CAMK2A and KHSRP could be related to nervous system-associated symptoms in patients with FD. CAMK2A is a multifunctional protein kinase involved in excitatory synapses and is central to synaptic plasticity. It has catalytic functions triggered by increased calcium ion concentration, as well as essential structural roles. It is highly expressed in the CNS accounting for 1–2% of total hippocampal proteins and up to 10% of the postsynaptic density [77,78]. KHSRP takes part of multi-protein complex participating in post-transcriptional control of gene expression (e.g., pre-mRNA splicing, mRNA decay, microRNA biogenesis etc.), particularly constitutes an enhancer element upstream of the c-myc oncogene promoter [79]. The scientific literature indicates that the pathogenesis of FD is even localized at the level of the CNS [14,17,20,26,27,28,80]. However, there is a notable lack of studies on repercussions beyond cerebral vasculopathy, such as synaptic dysfunction, and on long-term effects, such as depression, Parkinson’s disease, anxiety, and low quality of life, highlighting the need for more research in these areas [81]. From a pathophysiological point of view, lysosomal dysfunction can alter the composition of and interactions in the plasma membrane [82] and can suppress autophagy and mitochondrial function [83]. Therefore, lysosomal dysfunction could interfere with energy production and the maintenance of synaptic homeostasis. On the other hand, alterations in the release of neurotransmitters [84] (such as acetylcholine, dopamine, or serotonin) have been observed. Cholinergic neurons are associated with eccrine sweat glands in blood vessels, hair follicles, and cutaneous sensory nerve endings, and acetylcholine release may be associated with the neuropathic pain present in FD. Presynaptic dopaminergic alterations and an imbalance between cholinergic and dopaminergic activities in the CNS are present in patients with FD. According to the scientific literature, in patients with FD, there are alterations at the serotonergic system level. Changes in a broad spectrum of functions (such as mood, cognition, anxiety, learning, memory, reward processing, and sleep) have been observed. These disorders can lead to schizophrenia, mood disorders, and autism [85]. CAMK2A as previously noted, is involved in various cellular processes, including cell proliferation, synaptic plasticity, learning, and memory [78]. Its role in synaptic function is most likely related to the neurotransmitter dysfunction observed in FD [81]. Other hand, KHSRP is a highly expressed protein in neuronal cells [86], and it is involved in the immune cell function and homeostasis, inflammatory response, and cell proliferation [87]. A deficit in neuronal tissue expression of KHSRP is associated with impaired neuronal development, which, among other effects, results in altered synaptic transmission [88], as previously pointed out in FD neuronal pathophysiology. The resulting clinical manifestations derived from these four biomarkers that are proposed by the model could make clinical sense.

Various biomarkers depend on the nature of the detected molecule, such as a metabolite, DNA sequence, lipid, or protein [89]. One of the limitations of our study is that it was based on a proteomic model. We based our study on previous studies that obtained satisfactory results using machine learning-based technology [41,42,90]. Although we only used molecules of a protein nature, this study could be a good approximation. Notably, in the case of lysosomal diseases, such as FD, accumulated primary and secondary metabolites or proteins that are explicitly secreted by storage cells are good candidates for biomarkers [91].

Systems biology and computational modeling are good tools for investigating rare diseases [40], they have limitations. First, all modeling approaches are limited by the information about diseases, drugs, and biological data available in public repositories. The molecular definition of FD could be biased or incomplete owing to its wide range of manifestations. However, we were able to identify 92 proteins that are functionally involved in FD development through several cellular-level pathological processes (α-Gal A is absent or malfunctioning, and Gb3 accumulation is involved in oxidative stress, impaired autophagy, cellular death, lipid raft disruption, compromised energy metabolism, and inflammation and immune responses) and are associated with relevant tissue-level dysfunctions in FD, such as vascular dysfunction. We can highlight apoptosis-inducing factor mitochondria associated, nitric oxide synthases 2 and 3, intercellular adhesion molecule 1, vascular cell adhesion molecule 1, superoxide dismutase 2, interleukin-6, TNF-α, endothelial leukocyte adhesion molecule-1, and prostaglandin-endoperoxide synthases 1 and 2, which are associated with oxidative stress, inflammation, and cell death and, subsequently, with vascular dysfunction [25,92,93,94]. Furthermore, employing these models, we were able to propose newly described candidates related to both vascular and neuronal locations. Second, owing to their protein interaction-based nature, our models are limited to the evaluation of proteins. Thus, metabolites, lipids [95], or other measurable candidates could not be evaluated. While we focused on highlighting the most promising protein-based candidates, the complete list of candidates could be screened to find measurable proxies and obtain further biomarker candidates (Supplementary Table S3). Finally, our models reflect protein activity, and thus, the results might not directly translate to protein levels and, therefore, to clinical symptomatology. Experimental validation of these findings must consider the evaluation of different approaches, in addition to the quantification, such as functional or regulatory changes or even changes at the expression level.

From our standpoint, a potential in vitro/in vivo validation [96] of the prognostic and early detection potential of the candidates identified in this study would entail quantifying the concentrations of these biomarkers in plasma samples from newly diagnosed classic and atypical Fabry patients, stratified by gender. These biomarker concentrations would be correlated with a validated instrument for assessing overall disease severity and organ-specific domains, such as the Mainz Severity Score Index (MSSI) [97]. The plasma levels of the candidate biomarkers, along with the MSSI and their respective domains, would be measured at regular intervals throughout the study period until the patient commences specific treatment or experiences a clinical event related to Fabry disease.

Finally, the key is to have biomarkers that help provide more precise and early management of organ damage, particularly in late phenotypes, to conduct early therapeutic intervention and stabilize or slow down the appearance of irreversible comorbidities in patients. Although this was an in silico study, our methodology showed satisfactory results in deciphering the potential mechanism of action of some proteins involved in vascular events [46], as well as in nervous system morbidity [98]. This in silico study suggests more focused biomarkers to more accurately make evident vascular and neurological damage early, and therefore carry out early intervention in FD. Certainly, human studies with a representative patient cohort are required for validation [96]. However, this in silico modeling study presents promising preliminary results for biomarkers which are quantifiable from biological samples easily obtained in routine clinical practice, such as peripheral blood (plasma) and/or urine, suggesting that these biomarkers have excellent future clinical applicability for the early detection of damage in vascular and unexplored neuronal tissues and are not yet applied in routine practice.

## 4. Materials and Methods

**Figure 3:**
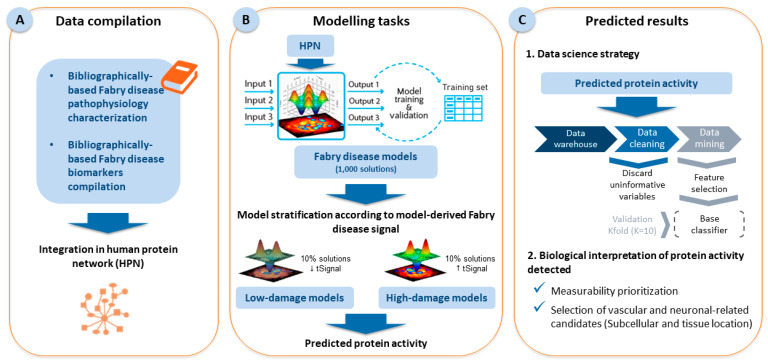
Scheme of the methodology followed in the present study for Fabry disease.

The in silico study was developed using the TPMS [42] based on systems biology, machine learning and pattern recognition techniques that integrate all available biological, pharmacological, and medical knowledge to create mathematical models that simulate pathology and human physiology. Figure 3 summarizes the steps followed in this study.

### 4.1. Generation of Mathematical Models

The first step for FD modeling was molecular characterization (Figure 3, Section A). We performed a nonsystematic full-length literature review of relevant articles in the PubMed^®^/Medline database. The search strategy is shown in Supplementary Table S5. For the articles obtained, the title, abstract, and full text were reviewed by two authors minimum, in case of doubt third reviewer opinion was gather. The references of the included articles were also checked. The objective of this study was to identify processes (motives) and proteins (effectors) that are functionally involved in FD (Table 3, Supplementary Table S1) and, on the other hand, already described biomarkers of FD, either clinically established or experimentally proposed (Supplementary Table S2).

This information search process has been applied in previous studies to create models leading to experimentally valid conclusions [45,46,47,48,98].

The TPMS technology, which has been extensively described elsewhere [42], was applied to create FD models based on protein–protein interaction networks (Figure 3, Section A), including information from dedicated databases (KEGG [99], REACTOME [100], INTACT [101], BIOGRID [102], HPRD [103], and TRRUST [104]). The human biological map included 9226 proteins and 83,011 relationships. Network representations were created using Cytoscape version 3.8 [49].

Models were trained using a compendium of biological and clinical data that describe human physiology (Supplementary Table S4) [105,106], defined as input–output relationships between drugs and condition definitions at the protein level, to obtain a multilayer perceptron (MLP) of an artificial neural network over the human protein network [41,42] (Figure 3, Section B). The modeling focused on molecular characterization of FD (Supplementary Table S1), and sampling methods were applied to describe plausible relationships between different regions of the network. These methods result in predicted mechanisms of action between a stimulus and a response composed of a universe of plausible solutions. The mean among these solutions provides the most likely path from the stimulus to the response. In addition to the interactions linking the stimulus and the response, the sampling-based methods models allow the exploration of the predicted activity for each protein [ranging between (−1, 1)] within the models and the exploration of how the signal flows among the proteins in the network.

To identify the FD effectors with greater potential to induce the disease, in addition to α-Gal A deficiency, and thus to be used as a stimulus in the model, the signal was propagated from each of the proteins under evaluation (e.g., each of the FD effectors) through the sampling-based methods models toward the response (e.g., the rest of the FD definition). The number of FD proteins that were stimulated, with the sign assigned in the characterization at distances 1, 2, and 3, was measured for each protein effector; protein proximity was positively pondered. A mean value was obtained for the universe of solutions, and a normalized value was obtained considering the maximum signal that could be achieved (maximum value: 100%). We selected as a stimulus the FD group of effectors that reached at least 75% of the rest of the effectors, together with α-Gal A deficiency.

A model containing 1000 solutions, which can be considered virtual individuals, was generated. Considering that each protein in the model has an associated predicted protein activity, the activity of protein sets can be quantified through the tSignal parameter, as previously described [37]; that is, the mean signal for the modulated proteins of a set:

(1) $${tSignal}_{prot\;set}=-\frac{1}{n}\sum_{i=1}^{n}v_{i}y_{i}\;$$

where n is the number of modulated proteins within the set, v refers to the signal value of protein i, and y is the original protein signal. This parameter was calculated for each of the 1000 solutions of the models and was considered a proxy of the number of cellular alterations associated with FD development. Thus, two cohorts were defined as follows: low-damage models (10% of solutions with a lower tSignal, e.g., those for which the stimulus induces less disease) and high-damage models (10% of solutions with a higher tSignal, e.g., those for which the stimulus induces more disease) (Figure 3, Section B). The predicted protein activity levels were extracted from each of the solutions in both cohorts for subsequent biomarker evaluation.

Although the TPMS models are protein-based, the interactome in which they are built includes gene and RNA regulation data; thus, for standardization purposes, we used gene names to refer to all genes/proteins in the model results mentioned in the tables and figures.

### 4.2. Statistical Analysis: Classifier Identification

A data science strategy (Figure 3, Section C.1) was applied to explore the potential of the predicted protein values (variables) to classify the models based on 3 steps: data cleaning, data mining, and cross-validation. Data cleaning consisted of removing uninformative variables. Data mining included two steps: First, feature selection was performed for the evaluation of classifiers composed of two variables (1-variable classifiers were explored by brute force), applying the following feature selection methods: CHOW-LIU [107], MRMR [108], RELIEFF [109], RFE-SVM [110], SFFS [111], and Wilcoxon with correlation [112]. Then, a base classifier was calculated through either optimal linear or quadratic threshold identification for classifiers composed of 1 variable or generalized linear model-binomial [113], naive Bayes [114], or MLPs for classifiers composed of 2 variables. Finally, 10 K-fold cross-validation was applied. This validation consists of the estimation of the threshold value for 10 random subsets of all available samples, and the final threshold is determined as the average of all the thresholds determined. Through this step, we selected candidates with a higher generalization capability. The cross-validated accuracy was used as a classifier optimization and quality measure, together with the cross-validated *p* value.

All simulations and computational processes described in this project were executed in the Anaxomics computing cloud, which integrates more than 800 computational threads in machines with 64 gigabytes of RAM. The software, databases, and tools used were from Anaxomics Biotech.

### 4.3. Biological Data Compilation and Filter Application for the Selection of Risk Biomarkers

To further filter the classifiers for obtaining the most promising biomarker candidates from biological and clinical points of view, we used information stored in dedicated databases (Figure 3, Section C.2). To obtain information on biomarkers detectable in plasma and urine, we explored The Human Protein Atlas (THPA) [52,53] and the Clinical Urine Proteomics Database [115]. We used the subcellular and tissue locations of the identified biomarkers retrieved from UniProtKB [51] and THPA [52,53] to determine their presence in vascular and neuronal tissues (Table 4) and to prioritize candidates related to alterations associated with these locations. We also performed a specific search for candidates related to the CNS to explore and identify potential biomarkers that could be involved in FD. From THPA, only “enhanced” or “supported” entries were considered. Finally, information on related metabolites according to the Human Metabolome Database [116] was retrieved.

## Figures and Tables

**Table 1:** Classification capabilities of previously proposed biomarkers according to our models. β-2-Microglobulin (highlighted with a gray background) was selected as the selection threshold.

Biomarker (Gene Symbol) [50]	Biomarker (UniProt Code) [51]	Cross-Validated Accuracy (%)	Cross-Validated *p* Value
*MAPK11*	Q15759	76	7.41 × 10^−20^
***B2M***	**P61769**	**71**	1.18 × 10^−10^
*CD80*	P33681	68	2.14 × 10^−7^
*CCL2*	P13500	66	4.77 × 10^−6^
*NOS3*	P29474	63	9.04 × 10^−6^
*GPX1*	P07203	61	3.54 × 10^−6^
*ACTB*	P60709	61	0.0000772
*TGFB1*	P01137	61	0.000801
*AGTR1*	P30556	60	0.004
*MMP9*	P14780	59	0.002

*ACTB*: β-actin; *AGTR1*: Angiotensin II receptor type 1; *B2M*: β-2-microglobulin; *CCL2*: C-C motif chemokine 2; *CD80*: Homo sapiens CD80 molecule; *GPX1*: Glutathione peroxidase 1; *MAPK11*: Mitogen-activated protein kinase 11; *MMP9*: Matrix metallopeptidase 9; *NOS3*: Nitric oxide synthase, endothelial; *TGFB1*: Transforming growth factor β-1.

**Table 2:** Biomarker candidates reported to be measurable in body fluids (plasma/urine) and present in neuronal and/or vascular locations. Entries highlighted with a gray background are presenting with a cross-validated accuracy of ≥90% and have been reported to be present in both neuronal and vascular locations.

Biomarker Candidate * (Gene Symbol) [50]	Biomarker Candidate (UniProt Code) [51]	Plasma Protein	Urine Protein	Cross-Validated Accuracy (%)	Location ** [51,52,53]
*CAMK2A*	Q9UQM7	YES	NO	100	NV
*CTSS*	P25774	YES	NO	100	N
*FOSL1*	P15407	YES	NO	100	N
*PPP1CC*	P36873	YES	NO	100	N
*ILK*	Q13418	YES	NO	99	NV
*CDK1*	P06493	YES	NO	96	N
*GNB5*	O14775	YES	NO	96	N
*AKT3*	Q9Y243	YES	NO	96	N
*LMNA*	P02545	YES	YES	95	NV
*MAPK1*	P28482	YES	YES	95	N
*KHSRP*	Q92945	YES	NO	93	NV
*PTPN13*	Q12923	NO	YES	92	N
*GNB3*	P16520	YES	NO	89	N
*STK3*	Q13188	YES	NO	88	N
*TGFB2*	P61812	NO	YES	88	N
*CIITA*	P33076	YES	NO	87	N
*ELAVL1*	Q15717	YES	NO	87	N
*VIM*	P08670	YES	NO	86	N
*NCAM1*	P13591	YES	YES	84	NV
*IL18*	Q14116	YES	YES	84	NV
*KPNB1*	Q14974	YES	NO	81	NV
*GRIN1*	Q05586	YES	NO	80	N
*APP*	P05067	YES	YES	78	N
*DNMT1*	P26358	YES	NO	78	N
*PML*	P29590	YES	NO	76	NV
*BRCA1*	P38398	YES	NO	76	NV
*TYMS*	P04818	YES	NO	75	NV
*SPP1*	P10451	YES	NO	75	N
*ANK3*	Q12955	YES	NO	74	N
*ETS1*	P14921	YES	NO	73	N
*PKN2*	Q16513	YES	NO	72	N
*YWHAQ*	P27348	YES	NO	72	N
*HIPK2*	Q9H2X6	YES	NO	71	NV
*ANK2*	Q01484	YES	NO	71	NV
*DCC*	P43146	YES	NO	71	N
*B2M*	P61769	YES	NO	71	NV

* The complete list of gene names can be found in Supplementary Table S3. ** N = neuronal; NV = neuronal and vascular.

**Table 3:** Motives identified by the Fabry disease bibliography-based molecular characterization. The “*Motive*” column allows this table to be linked to Supplementary Table S1.

Motive	Motive Name	Proteins
1	α-Galactosidase A mutation and Gb3 accumulation	13
2	Oxidative stress	16
3	Impaired autophagy	8
4	Cellular death	10
5	Lipid raft disruption	4
6	Compromised energy metabolism	17
7	Inflammation and immune response	27

**Table 4:** Subcellular location and tissue terms considered for neuronal- and vascular-related proteins in each database.

		Neuronal-Related Proteins	Vascular-Related Proteins
**Subcellular location**	THPA [52,53]	-	-
	UniProt [51]	axon, dendrite, midbody, synaptic vesicle membrane, neuronal cell body, axon initial segment, terminal bouton, dendritic spine, postsynaptic density of dendrite, neuron projection, presynaptic membrane, synapse, postsynaptic membrane, cell body, synaptic cleft, myelin sheath, synaptic vesicle, paranode region of axon, dendritic branch, excitatory synapse	-
**Tissue**	THPA [52,53]	caudate, cerebellum, cerebral cortex, hippocampus	smooth muscle
	UniProt [51]	cerebrospinal fluid, peripheral nerve, pituitary, substantia nigra, temporal cortex, glial cell, brain stem, hippocampus, cerebellum, nervous system, neuron, astrocyte, Schwann cell, nerve, brain	vascular smooth muscle, smooth muscle cell, smooth muscle, endothelial cell

## Data Availability

The original contributions presented in the study are included in the article and Supplementary Materials, further inquiries can be directed to the corresponding author.

## Supplementary Materials

The following supporting information can be downloaded at https://www.mdpi.com/article/10.3390/ijms251910329/s1. Reference [117] has been mentioned in Table S2.
